# Fertilization in *C. elegans *requires an intact C-terminal RING finger in sperm protein SPE-42

**DOI:** 10.1186/1471-213X-11-10

**Published:** 2011-02-23

**Authors:** Luke D Wilson, Jacqueline M Sackett, Bryce D Mieczkowski, Abigail L Richie, Kara Thoemke, Jon N Rumbley, Tim L Kroft

**Affiliations:** 1 Department of Biology, Swenson College of Science and Engineering, University of Minnesota Duluth, Duluth, MN 55812 USA; 2 Department of Biology, College of St. Scholastica, Duluth, MN 55811 USA; 3 Department of Pharmacy Practice and Pharmaceutical Sciences, College of Pharmacy, University of Minnesota Duluth, Duluth, MN 55812 USA

## Abstract

**Background:**

The *C. elegans *sperm protein SPE-42, a membrane protein of unknown structure and molecular function, is required for fertilization. Sperm from worms with *spe-42 *mutations appear normal but are unable to fertilize eggs. Sequence analysis revealed the presence of 8 conserved cysteine residues in the C-terminal cytoplasmic domain of this protein suggesting these residues form a zinc-coordinating RING finger structure.

**Results:**

We made an *in silico *structural model of the SPE-42 RING finger domain based on primary sequence analysis and previously reported RING structures. To test the model, we created *spe-42 *transgenes coding for mutations in each of the 8 cysteine residues predicted to coordinate Zn^++ ^ions in the RING finger motif. Transgenes were crossed into a *spe-42 *null background and protein function was measured by counting progeny. We found that all 8 cysteines are required for protein function. We also showed that sequence differences between the C-terminal 29 and 30 amino acids in *C. elegans *and *C. briggsae *SPE-42 following the RING finger domain are not responsible for the failure of the *C. briggsae *SPE-42 homolog to rescue *C. elegans spe-42 *mutants.

**Conclusions:**

The results suggest that a *bona fide *RING domain is present at the C-terminus of the SPE-42 protein and that this motif is required for sperm-egg interactions during *C. elegans *fertilization. Our structural model of the RING domain provides a starting point for further structure-function analysis of this critical region of the protein. The C-terminal domain swap experiment suggests that the incompatibility between the *C. elegans *and *C. briggsae *SPE-42 proteins is caused by small amino acid differences outside the C-terminal domain.

## Background

The union of sperm and egg pronuclei to form a new organism is the endpoint of a carefully choreographed process that includes gamete recognition, binding, and fusion of plasma membranes. Our understanding of the molecular processes underlying these phenomena has been shaped by studies in sea urchins [1], *Chlamydomonas *[2-4], *Drosophila *[5], mice [6-8] and, within the last decade, the nematode *Caenorhabditis elegans *[9-12]. *C. elegans *is particularly suited to the discovery of molecules necessary for spermatogenesis or fertilization because its hermaphroditic mode of reproduction is unique among genetic model organisms. Spermatogenesis defective (*spe*) mutants discovered in genetic screens using hermaphrodites can be easily recovered by mating wild type males into the sterile hermaphrodites.

Mutations in the *C. elegans spe-42 *gene result in the production of morphologically normal spermatozoa that are fertilization defective despite making direct contact with eggs in the spermatheca, the site of fertilization [13]. Six other *C. elegans *genes that have the same mutant phenotype as *spe-42 *constitute the *spe-9 *class [9-12,14], named for the first of these genes to be cloned [15]. The five cloned genes in this class have been shown, or are predicted, to be sperm plasma membrane proteins [13,16-19]. The phenotype of these mutants suggests that *spe-42 *and the other *spe-9 *class genes function at the moment that sperm and egg plasma membranes meet.

**Figure 1:**
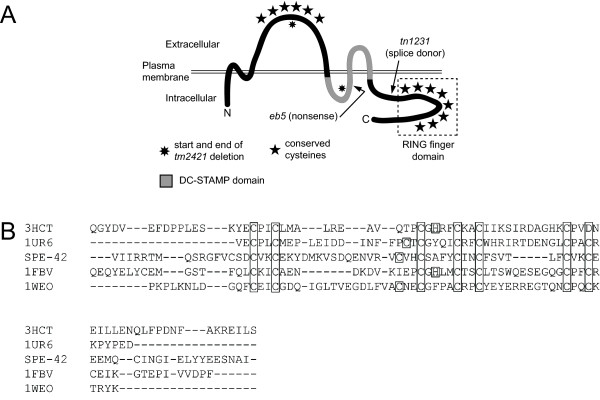
**SPE-42 membrane topology and sequence alignment of putative RING finger domain with known RING finger proteins**. (A) SPE-42 is predicted to be a 6-pass integral membrane protein with both N- and C-termini in the cytoplasmic space. Domains of interest include a large extracellular domain between transmembrane segments 3 and 4 containing 6 conserved cysteine residues, a DC-STAMP domain and a C-terminal cytoplasmic domain with a predicted RING finger. The locations of the *eb5 *and *tn1231 *point mutations and the *tm2421 *deletion are indicated. The *tm2421 *mutant is completely sterile at all temperatures (our unpublished data). (B) Amino acid sequence alignment of the RING finger domains (dashed box from panel A) of SPE-42 and the four proteins used to build the structural model. Two Zn^++ ^ions are predicted to be coordinated through electron sharing with the boxed residues shown in each protein. Structurally determined and putative SPE-42 Zn^++ ^liganding residues are boxed. N- and C-terminal amino acids were removed to optimize the alignment in PROMALS3 D. The misaligned Zn^++ ^ligand is the result of topologically equivalent displacement in the known structures dependent on amino acid identity, *i.e*. cysteine or histidine.

The consensus of 11 topology prediction algorithms [20-30] suggests that SPE-42 is a six-pass transmembrane protein with its N- and C-termini facing the cytosol (Figure 1A). Amino acid sequence analysis showed 3 domains of potential importance for SPE-42 protein function: (1) a large extracellular domain between transmembrane helices 3 and 4 containing six conserved cysteines separated by 9-13 amino acids, (2) a DC-STAMP domain [31] that includes transmembrane helices 5 and 6 and (3) a predicted RING finger [32,33] formed by 8 conserved cysteines in the C-terminal cytoplasmic domain. These 3 features are conserved in each of the 2 SPE-42 homologs that are present in many metazoan genomes including *Drosophila*, mice and humans [5, our unpublished data]. One of the two *Drosophila *SPE-42 homologs, Sneaky, is necessary for the plasma membrane breakdown (PMBD) event that occurs after a spermatozoon is engulfed by the egg during fertilization [5]. Mutation of the second cysteine in the large extracellular domain disrupts PMBD, indicating its importance for Sneaky function. Because it is predicted to be extracellular, this domain could potentially interact with proteins on the egg surface. The presence of a DC-STAMP domain, in both Sneaky and SPE-42, suggests SPE-42 may be involved in a membrane fusion event because the canonical DC-STAMP protein is required for cell fusion events in the mammalian monocyte cell lineage [34-36]. The mechanism through which DC-STAMP mediates membrane fusion is not presently clear.

SPE-42 homologs in all species examined to date possess a predicted RING finger motif near the C-terminus. RING fingers are structural domains, held together by the coordination of two Zn^++ ^ions by the side chains of nearby cysteine, histidine or aspartic acid side chains [32,37]. These motifs are most commonly found in E3 ubiquitin protein ligases [38,39] where they facilitate ubiquitination of target proteins [40]. E3 RING finger proteins simultaneously interact with a substrate and a ubiquitin-conjugated E2 enzyme, allowing transfer of ubiquitin to the substrate by the E2. Unlike HECT domain E3 s that covalently bind ubiquitin and transfer it to the substrate directly [41], RING domain E3 s lack catalytic activity. Although the SPE-42 protein does not show significant sequence homology to E3 ligases outside the RING finger, this domain is the chief unifying feature in an otherwise diverse E3 ligase family. Therefore, we cannot confirm or rule out an E3 ligase-like function for SPE-42. Sequence analysis of SPE-42 family members predicts that in all cases the Zn^++ ^ions are coordinated by eight cysteines (C4C4 pattern), unlike most E3 RING fingers, which include a histidine in a C3HC4 arrangement [39].

In the experiments described here, we used a systematic mutagenesis approach to characterize the cytoplasmic C-terminal region of SPE-42 containing the putative RING finger. The ability of *spe-42 *transgenes bearing single amino acid substitutions or larger scale changes to produce progeny in an otherwise self-sterile *spe-42 *null mutant strain allowed us to determine the importance of individual amino acids for SPE-42 function. Our results showed that the 8 cysteine residues predicted to form a RING finger are critical for SPE-42 function *in vivo*. We used these data along with previously solved RING finger structures to develop a structural model of the SPE-42 RING domain including predictions of the specific amino acids that are most likely to participate in protein-protein interactions.

## Results and discussion

### A structural model of the SPE-42 RING finger

The RING finger at the C-terminus of SPE-42 was predicted by primary amino acid sequence analysis and closely matches the consensus Zn^++ ^ligand spacing of the Pfam RING finger consensus (http://pfam.janelia.org/; accession number PF00097) and the cloned SPE-42 homolog Sneaky [5]. We developed a structural model of SPE-42 using the structures of known RING finger containing proteins. The model is based on sequence conservation with the four most closely related RING finger sequences in the RSCB protein data bank (PDB) http://www.rcsb.org/pdb/home/home.do; c-Cbl proto-oncogene RING domain [pbd:1FBV] [42], tumor necrosis factor receptor-associated factor-6 (TRAF-6) RING domain [pdb:3HCT] [40], CCR4-NOT transcription complex, subunit 4, (CNOT4) [pdb:1UR6] [43], and cellulose synthase, catalytic subunit (IRX3), [pdb:1WEO] (He, F., *et al*. personal communication). The criteria for selecting these four structures were overall sequence identity, number of contiguous homologous residues (SPE-42 sequence to known structure) and relative positioning/spacing of the zinc ligands. Multiple sequence alignment of the 5 RING domains resulted in 7 of 8 ligands to the two zinc centers aligning across all sequences represented (Figure 1B). Based on the known structures, we observed topological flexibility about ligands 3 and 4 leading to the misalignment of the last zinc ligand. Nonetheless, the overall RING finger domain fold was maintained (Additional file 1: panel A). Additional file 1: panel B shows the homology model of SPE-42 based on the four RING finger structures. Importantly, polar and charged residues were found in more solvent exposed positions while non-polar residues were less solvent exposed in our homology model.

**Figure 2:**
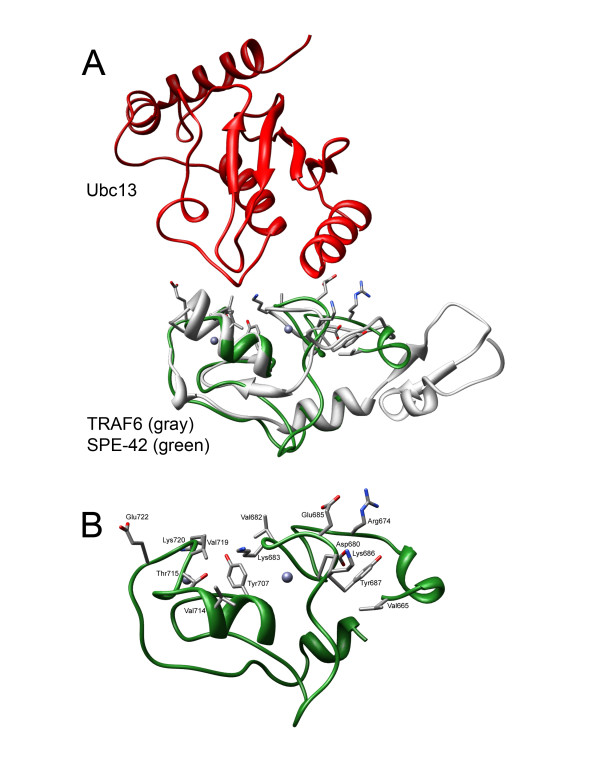
**Putative protein-protein contact residues in SPE-42 homology model**. (A) Interaction between TRAF6 (gray) and Ubc13 (red) including the SPE-42 RING domain (green) aligned for comparison. TRAF-6 was co-crystalized with Ubc13 in pdb:3HCT. Following structural alignment of SPE-42, all residues within 5Å of Ubc13 were determined and shown. (B) SPE-42 RING domain alone with putative protein-protein interacting residues on SPE-42 surface shown. Individual amino acids are labelled and numbered in accordance with the complete SPE-42 amino acid sequence.

**Figure 3:**
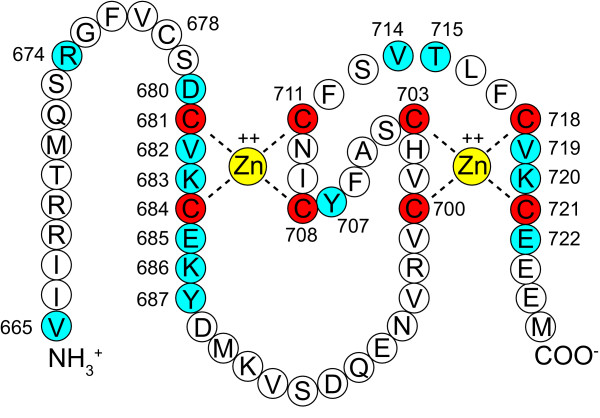
**RING domain amino acids with protein-protein interaction potential**. The 8 cysteines shown in red are predicted to coordinate the two Zn^++ ^ions, stabilizing the RING finger structure. They are indispensable for SPE-42 function except for C681, loss of which can be partially compensated for by C678 (see Tables 1 and 2). Amino acids colored blue are predicted to be on the protein surface and within 5Å of a theoretical binding partner by the model shown in Fig. 2B.

Because RING finger domains are typically protein-protein interaction sites, we identified the potential protein-protein interaction surface and residues on the SPE-42 model. The template structure 3HCT contains both the TRAF-6 RING finger domain and its interaction partner Ubc13, providing a means to assess critical points of contact between them. Figure 2 shows the structural alignment of SPE-42 and TRAF-6 RING finger domains with respect to TRAF-6 binding partner Ubc13. All residues in SPE-42 within 5Å of Ubc13 resulting from the alignment are shown. Figure 3 shows the RING finger sequence with these residues colored blue. The sequential location of identified residues with respect to the Zn^++ ^ligands is consistent with well-characterized E2 ubiquitin conjugating-E3 ubiquitin ligase interaction sites [39].

An additional outcome of the SPE-42/RING finger protein alignment and homology model generation was the exclusion of the first cysteine, C678, in this short sequence domain from participating in metal binding. Further, the proximity of cysteine 678 to cysteine 681, both in sequence space and in the SPE-42 model, led to the prediction that mutation of cysteine 681 would show an intermediate, non-null, phenotype. This would be expected if cysteine 678 is able to rearrange and weakly associate with the Zn^++ ^in place of cysteine 681. An extension of this prediction is that if both cysteine 678 and cysteine 681 are mutated simultaneously the phenotype will follow that shown by the other cysteine residues in which Zn^++ ^binding is irreconcilably disrupted.

### Contribution of RING finger cysteines to SPE-42 protein function

Transgenic constructs coding for cysteine-to-alanine substitutions at each predicted RING finger cysteine (Figure 3, red amino acids) were created. Individual transgenes were crossed into a *spe-42(tn1231) *or a *spe-42(tm2421) *genetic background and protein function was measured by counting live progeny. Worms homozygous for either of these *spe-42 *mutations are completely sterile at the 25°C assay temperature [13, Table 1], so any progeny produced result from transgenic rescue. The results of the crosses are reported in Table 1. SPE-42 protein function was severely reduced for all cysteine to alanine mutations except for cysteine 681. Surprisingly, average broods for the cysteine 681 mutation were 45% of broods from lines with a wild type transgene, suggesting that either this amino acid is not essential for Zn^++ ^coordination or some other amino acid was partially compensating for its loss. As predicted by our SPE-42 homology model above, a good candidate for the compensating amino acid was cysteine 678.

**Table 1:** Transgenic rescue with substitution mutations in RING finger cysteines.

Transgene	Mutation	Progeny	n
xyEx186	C681A	39 ± 6	10
xyEx187	C681A	51 ± 11	7
xyEx188	C681A	63 ± 12	5
			
xyEx69	C684A	6 ± 2	9
xyEx72	C684A	<1	6
xyEx77	C684A	0	9
			
xyEx48	C700A	<1	8
xyEx52	C700A	1 ± 0.68	8
xyEx63	C700A	<1	10
			
xyEx134	C703A	3 ± 1	12
xyEx135	C703A	<1	9
xyEx136	C703A	3 ± 1	10
			
xyEx73	C708A	0	5
xyEx78	C708A	0	5
xyEx80	C708A	0	5
			
xyEx74	C711A	<1	9
xyEx81	C711A	3 ± 1	10
xyEx129	C711A	2 ± 0.76	6
			
xyEx130	C718A	0	9
xyEx131	C718A	<1	9
xyEx132	C718A	0	9
			
xyEx85	C721A	2 ± 0.60	9
xyEx86	C721A	5 ± 2	10
xyEx87	C721A	11 ± 1	9
			
ebEx498	wild type	92 ± 16	11
xyEx175	wild type	123 ± 15	10
xyEx177	wild type	115 ± 17	11
			
N2 (wild type)	-	185 ± 5	10
			
spe-42(tn1231)	-	0	24
spe-42(tm2421)	-	0	15

Live progeny were counted for three independently-derived transgenes for each amino acid substitution. All transgenes are in a *spe-42(tn1231 or tm2421) *null genetic background. N2 and *spe-42 *worms listed at the bottom do not carry transgenes. All counts were conducted until the first day each worm produce no live progeny. Counts shown are ±SEM and were rounded to the nearest whole number with the exception of SEM values less than one. *spe-42(tn1231) *data are from [13].

To test this model, two additional transgenes were made: one in which cysteine 678 was substituted with alanine and a second in which both cysteines 678 and 681 were substituted (Table 2). As predicted by the model, transgenes with the cysteine 678 mutation rescued at the same level as wild type transgenes, indicating that this mutation alone does not affect protein function. The cysteine 678/681 double mutant reduced brood counts to the same low level observed for the other 7 cysteine mutant transgenes described above. These results clearly demonstrate that, while cysteine 678 is not required for Zn^++ ^coordination under normal circumstances, it can partially compensate for the loss of cysteine 681, restoring protein function to a level sufficient for fertilization to take place. Taken together, our results make a strong case that the 8 red cysteines shown in Figure 3 are actually involved in the Zn^++ ^coordination that holds together the SPE-42 RING finger structure and that our SPE-42 homology model, generated from known RING finger structures, is robust and predictive.

**Table 2:** Cysteine 678 is not part of the RING finger.

Transgene	Mutation	Progeny	n
*xyEx208*	C678A	117 ± 22	10
*xyEx209*	C678A	101 ± 22	9
*xyEx212*	C678A	113 ± 13	8
			
*xyEx186*	C681A	39 ± 6	10
xyEx187	C681A	51 ± 11	7
xyEx188	C681A	63 ± 12	5
			
*xyEx207*	C678A; C681A	3 ± 1	10
*xyEx213*	C678A; C681A	2 ± 1	10
*xyEx220*	C678A; C681A	1 ± 0.65	10
			
*ebEx498*	wild type	92 ± 16	11
*xyEx175*	wild type	123 ± 15	10
*xyEx177*	wild type	115 ± 17	11
			
N2 (wild type)	-	185 ± 5	10
			
*spe-42(tn1231)*	-	0	24
*spe-42(tm2421)*	-	0	15

Counts were conducted as described for table 1.

### Incompatibility of the *C. elegans *and *C. briggsae *SPE-42 proteins is not caused by the specific amino acid sequence C-terminal to the RING finger

**Figure 4:**
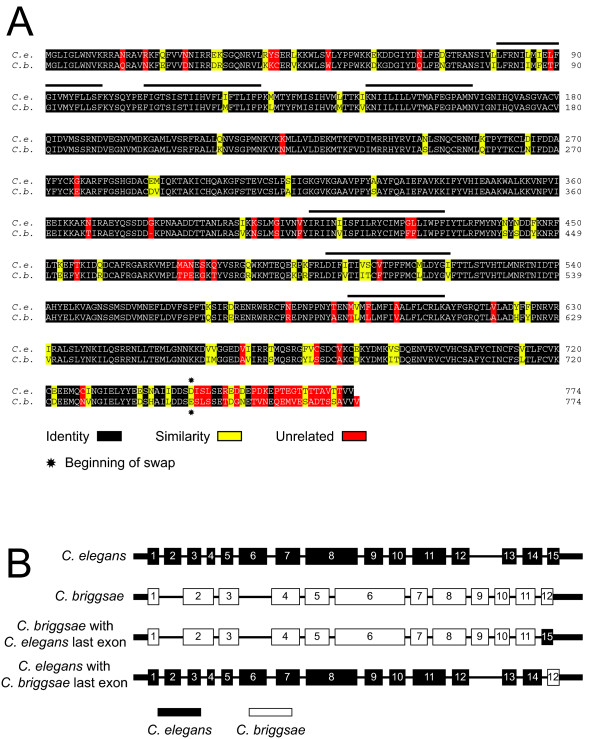
***C. elegans */*C. briggsae *chimeric constructs**. (A) Pair-wise comparison of *C. elegans *and *C. briggsae *SPE-42 proteins showing the striking divergence between the two proteins at the C-terminus. The divergent sequence corresponds exactly to the 3'-most exon of each gene. Solid lines above the sequence indicate membrane-spanning domains. (B) Chimeric constructs were made to determine the effect of the divergent sequence on protein function. All four constructs are under the control of the *C. elegans spe-42 *promoter and 3' UTR.

*C. briggsae *is in the closest sister taxon to *C. elegans*, the two species having diverged about 100 million years ago [44]. Although *C. elegans *and *C. briggsae *are difficult to distinguish by phenotype, they are reproductively isolated. Pair-wise comparison of *C. elegans *SPE-42 with its *C. briggsae *homolog revealed that the two proteins are 85% identical and 93.4% similar except for the C-terminal 29 and 30 amino acids, respectively, where they are only 25% identical and 37.5% similar [13, Figure 4A]. The divergent sequence is encoded by the last exon of each gene. The two most likely explanations for this divergence are first, that this part of the protein is not important for function and therefore is under very little evolutionary constraint or second, that the sequence changes define a key difference between the two proteins that may explain the observed reproductive isolation. A transgene containing a genomic DNA clone of the *C. briggsae spe-42 *homolog under the control of the *C. elegans spe-42 *promoter and 3' UTR (Figure 4B) failed to produce more than a few progeny in *spe-42 *null mutant worms, suggesting that some aspect of the *C. briggsae *protein is incompatible with the *C. elegans *fertilization machinery (Table 3). Two chimeric constructs in which the last exon of *C. elegans spe-42 *was swapped into the *C. briggsae *gene and vice versa were created to ask whether adding the *C. elegans *exon to the *C. briggsae *gene would improve transgenic rescue and adding the *C. briggsae *exon to the *C. elegans *gene would decrease progeny compared to a wild type *C. elegans spe-42 *transgene (Figure 4B). The results of these crosses show that replacing the C-terminal 30 amino acids in the *C. briggsae *protein with the C-terminal 29 amino acids in the *C. elegans *protein increased average progeny counts by only a few worms, while the reciprocal swap of *C. briggsae *amino acids into the *C. elegans *protein did not change progeny counts at all (Table 3). These results demonstrate that the incompatibility between the *C. elegans *and *C. briggsae *homologs therefore lies in one or more of the individual sequence changes scattered throughout the rest of the protein. These differences define a starting point for continuing our structure-function investigation of SPE-42 outside of the RING finger domain.

**Table 3:** *C. elegans */*C. briggsae spe-42 *final exon swap.

Construct	Transgene	Progeny	n
	*ebEx498*	92 ± 16	11
	*xyEx175*	123 ± 15	10
	*xyEx177*	115 ± 17	11
			
	*xyEx1*	2 ± 1	10
	*xyEx2*	1 ± 0.62	9
	*xyEx3*	6 ± 2	9
			
	*xyEx27*	11 ± 4	10
	*xyEx30*	8 ± 4	10
	*xyEx34*	17 ± 4	10
			
	*xyEx36*	83 ± 15	9
	*xyEx37*	110 ± 10	8
	*xyEx41*	123 ± 15	10
			
	N2 (wild type)	185 ± 5	10
			
	*spe-42(tn1231)*	0	24
	*spe-42(tm2421)*	0	15

Transgenic rescue was quantified for three independently-derived transgenes for each construct. Exon shading scheme is the same as in Fig. 4B. Constructs were fused to the *C. elegans *promoter and 3' UTR at the start and stop codons. All transgenes are in a *spe-42 *null genetic background. N2 and *spe-42 *worms listed at the bottom do not carry transgenes. All counts were conducted until the first day each worm produced no live progeny. Counts are ± SEM rounded to the nearest whole number except for values less than one. The *spe-42(tn1231) *data are from [13].

### Potential function of the RING domain

RING fingers are multi-functional protein-protein interaction domains normally associated with E3 ubiquitin ligase activity. Although examples of RING fingers in proteins that are not E3 s may exist, functional data support an E3 ligase activity for almost half of the 300 predicted human RING finger proteins [39]; the rest either have not been examined or no alternative molecular mechanism of action has been assigned. Some RING finger proteins like Bard1 do not possess intrinsic E3 ligase activity but are instead part of a multi-protein complex that does, in this case the Brca1-Bard1 heterodimer [45]. Other RING fingers like TRAF6 must form homodimers in order to interact with E2 ligases [40].

The SPE-42 RING finger could potentially function in multiple ways during *C. elegans *fertilization. Regardless of whether SPE-42 is itself an E3 ubiquitin ligase, is part of an E3 complex with other proteins, or does not have E3 activity at all, the RING finger-like domain likely mediates protein-protein interactions. SPE-42 interaction with its binding partner(s) could serve as a checkpoint for sperm competence to fuse with the egg, as a signal to the egg that the sperm nucleus and other contents have been delivered, or even as a signal that fusion has occurred and membrane proteins important for fertilization should be degraded to prevent polyspermy. Recent evidence from *Chlamydomonas *showed that FUS1 and HAP2, proteins essential for gamete fusion, are rapidly degraded immediately following membrane fusion [2]. These researchers further demonstrated that it was the membrane fusion event and not merely gamete binding prior to fusion that initiated protein degradation. The SPE-42 RING finger could act while still tethered to the plasma membrane or it could be cleaved from the rest of the protein, releasing it into the cytoplasm to act elsewhere. The mouse RING finger protein 13 (RNF13) is an E3 ubiquitin ligase that is normally an integral endosomal membrane protein. RNF13 can be proteolytically cleaved, however, releasing the RING domain into the cytoplasm [46]. SPE-42 also has a DC-STAMP domain that may be involved in membrane fusion events and a large extracellular domain that could interact with egg surface proteins. SPE-42 therefore has potential to interact with egg surface proteins, help to mediate sperm-egg fusion and signal that fusion has occurred.

### Use of SPE-42 expressed from extrachromosomal transgenic arrays to analyze protein function

Extrachromosomal arrays composed entirely of simple repeats of the injected experimental and marker DNA are selectively targeted for silencing in the male germline [47,48]. The presumably small amount of protein expressed from simple-repeat extrachromosomal arrays is not detectable by immunofluorescence or western blot using antibodies that can detect both wild type and mutant protein expressed from the endogenous gene locus [17]. Nevertheless, this small amount of protein is sufficient to produce robust rescue of every cloned *spe *and *fer *mutant described in the literature [for individual references, see 9, 12].

Hemagglutinin (HA) epitope-tagged SPE-42 expressed from a low-copy genomic insertion is not detectable by a commercial HA monoclonal antibody despite the fact that the transgene rescues the self-sterile Spe-42 mutant phenotype (H. Nishimura and S.W. L'Hernault, personal communication). Attempts by the same group to detect a SPE-42::HA fusion expressed in CHO tissue culture cells have also been unsuccessful. Because the mutant SPE-42 protein expressed in our assays is not detectable, the possibility that the loss of fertility observed with the mutant transgenes is due to protein degradation or mis-localization instead of a simple loss of protein function cannot be excluded. The complete rescue observed in the C678A mutant, partial rescue seen in the C681A mutant and almost complete lack of rescue seen in the C678A; C681A double mutant strongly suggest that C678 can at least partially compensate for loss of C681 but that C681 is actually involved in Zn^++ ^coordination in wild type SPE-42. Our results conclusively demonstrate that enough functional protein is reaching the site of action to provide ~46% of wild type activity levels in the C681A mutant, suggesting that significant degradation is not occuring. It is therefore plausible that SPE-42 protein expressed from our mutant transgenes is correctly localized and simply lacks function.

## Conclusions

We built a structural model of the SPE-42 RING finger domain based on solved structures of other RING domain proteins. Our experimental results demonstrate that 8 cysteine residues predicted to form a RING finger in the C-terminal domain of SPE-42 by the model are critical for protein function during fertilization. We also showed that incompatibility of the *C. elegans *and *C. briggsae *SPE-42 homologs does not result from evolutionary divergence in amino acid sequence at the C-terminus of the proteins. Our data provide a starting point for further investigation of SPE-42 function during *C. elegans *fertilization and elucidation of the potential roles of SPE-42 homologs in other species.

## Methods

### Worm strains and handling

Worm culture and genetic crosses were performed according to standard methods [49]. Bristol N2 was used as the wild type strain, and all mutants are in an N2 genetic background. The following mutant alleles, markers and genetic balancers were used: *him-8(e1489)*IV [50], *nT1[qIs51]*(IV;V) [51, K. Siegfried and J. Kimble, personal communication], *spe-42(tn1231)*V [13], *spe-42(tm2421) *V (S. Mitani, personal communication), *mIs10*V (K. Liu and A. Fire, personal communication).

### DNA constructs

A 4946 bp *spe-42 *genomic DNA fragment of cosmid B0240, pTK15, was created by sequentially cloning PCR products made with primers TK306 and TK309 (5'-GCGGGCCCTGAAACAATAAATCAGTGAATTAG-3'; 5'-GTCTCGAGTTGACTGAAATATTTTCAATTCCTCG-3'; 944 bp; ApaI and XhoI restriction sites), TK310 and TK311 (5'-AACTCGAGACCGGAAATTCCCATTTACC-3'; 5'-GTTCTAGATGAAAAGATGACAAAGTAAGTTG-3'; 1635 bp; XhoI and XbaI restriction sites) and TK312 and TK313 (5'-GGCCACCGCGGTGGCGAGTTTGTGGTTTC-3'; 5'-CATCTAGAACCAATAGCATTTTCTTGACC-3'; 2367 bp; XbaI and BstXI sites) into pBluescript II SK(+) (Agilent Technologies). During PCR, the central 6 bases in the *spe-42 *BstXI site were changed to match those in the plasmid. Pfu Turbo polymerase (Agilent Technologies) was used for PCR, and all products were sequenced. This construct (pTK15; "wild type" in Tables 1, 2 and 3; "*C. elegans*" in Figure 4B) includes 1409 bp of promoter sequence and 265 bp of 3' UTR sequence in addition to the *spe-42 *coding region and contains no genes other than *spe-42 *(B0240.2). Mutations to create cysteine to alanine substitutions in the SPE-42 RING domain were made using an overlap PCR strategy and cloned into the ApaI and XhoI sites in pTK15, replacing the wild type sequence. One of the 2 *C. elegans *preferred alanine codons (GCT or GCC) was used for all mutations to ensure robust protein expression [52]. Primer sequences used to make mutations are available upon request.

A 6078 bp genomic DNA fragment containing the *C. briggsae spe-42 *homolog was amplified from *C. briggsae *genomic DNA using the Expand Long Template PCR System (Roche) with primers TK330 (5'-GCCTCGAGTGAATGTTAATGAGCAGCCACC-3') and TK331 (5'-GCGGATCCGTGGGATGGTGGAAGCAGAAAGTTG-3') and cloned into the BamHI and XhoI sites of pBluescript II SK(+) to create pTK14. Overlap PCR using amplimers made with primers T7 and TK361 (5'-GTAATACGACTCACTATAGGGC-3'; 5'-GTTCTGCAGTTGTTGTTTGAATGATGTTTGAAAAATTAACTTTC-3') and primers TK360 and TK412 (GAAAGTTAATTTTTCAAACATCATTCAAACAACAACTGCAGAAC-3'; 5'-GCCTCGAGAAATGCGGCAGACGATAC-3') was used to join the *C. briggsae *gene 3' exons to the *C. elegans *stop codon and 3' UTR. The 1775 bp amplimer was digested with ApaI and XhoI and cloned into the corresponding sites of pTK15 to make pTK43. Overlap PCR using amplimers made with primers T3 and TK343 (5'-CGCAATTAACCCTCACTAAAGGG-3'; 5'-CATAATCCTATCAACCCCATAGTTGATACAATTCAGTTAGATTTTA-3') and primers TK342 and TK359 (5'-TAAAATCTAACTGAATTGTATCAACTATGGGGTTGATAGGATTATG-3'; 5'-GCTCGAGGTTTCAAACATGATGATGTTCCGGA-3') was used to join the *C. elegans spe-42 *promoter to the *C. briggsae *gene at the start codon. A 1949 BstXI/XhoI fragment of the amplimer was cloned into pTK43 to create pTK44. The central part of the *C. briggsae spe-42 *gene was cut from pTK14 with BspEI and ClaI and cloned into pTK44 to make pTK45 ("*C. briggsae*" in Figure 4B). The pTK45 construct contains the entire *C. briggsae spe-42 *gene (including introns) under the control of *C. elegans *5' and 3' regulatory sequence. Overlap PCR using amplimers made with primers T7 (sequence above) and TK413 (5'-TTTTGTGACTAAATTTAATTTCAGATATTTCATTGAGTGAACGC-3') and primers TK414 and TK345 (5'-GCGTTCACTCAATGAAATATCTGAAATTAAATTTAGTCACAAAA-3' and TAGAGTTCGTGCTCTTAGCCTGTAC-3') was used to join the last exon of *C. elegans spe-42 *to the penultimate exon of the *C. briggsae *gene. This 882 bp chimeric fragment was cloned into the KpnI and XbaI sites of pTK45 to create pTK46 ("*C. briggsae *with *C. elegans *last exon" in Figure 4B). In the reciprocal swap, overlap PCR using amplimers made with primers T7 and TK415 (5'-AACATTAATAATCATCTATTCCAGAAAGTTTATCTAGTGAAACAG-3') primers TK309 (sequence above) and TK416 (5'-CTGTTTCACTAGATAAACTTTCTGGAATAGATGATTATTAATGTT-3') was used to join the *C. briggsae *last exon to the penultimate exon of the *C. elegans *gene. The 982 bp fragment was cloned into the ApaI and XhoI sites of pTK15 to make pTK47 ("*C. elegans *with *C. briggsae *last exon" in Figure 4B).

### Transgenes and fertility assays

Transgenes were created by co-injecting *spe-42 *DNA constructs with the marker plasmid pPD118.20 (Fire Lab Vector Kit), which drives GFP expression in body wall muscle under the control of the *myo-3 *promoter using standard protocols [53]. At least 3 independently derived transgenes were analyzed for each mutant construct to control for variations inherent to extrachromosomal arrays. Transgenes were crossed into a *spe-42(tn1231 or tm2421) *mutant background using *myo-3::GFP *to follow the transgenes and *mIs10*, an integrated *myo-2::GFP *transgene, to pick *spe-42(tn1231 or tm2421) *homozygotes. Crosses were conducted at 20°C to facilitate efficient mating until the last step when *spe-42(tn1231 or tm2421)*/*mIs10; xyEx *mutant transgene hermaphrodites were transferred to 25°C. Worms used for progeny counts were therefore raised exclusively at 25°C since they were embryos. Crossing over between *mIs10 *and *spe-42 *was occasionally observed. To control for this possibility, non-GFP worms were picked from suspected recombinants and tested for fertility. Lack of complete sterility of *spe-42(tn1231 or tm2421) *homozygotes at the 25°C assay temperature is an indicator for recombination. Broods from recombinant worms were excluded from analysis. Transgenic worms were picked to individual NGM plates at 25°C and transferred to new plates daily. Counts for each worm were continued until the first day in which no live progeny were produced.

### SPE-42 homology model generation

The relationship of the SPE-42 c-terminal domain to RING finger containing proteins was determined by BLAST search with a low stringency expect threshold [54]. To generate a homology model, all RING finger containing structures in the RSCB protein data bank (PBD) were extracted. Sequences were retrieved by common name, sequence similarity to known RING fingers, and structure search using the VAST algorithm [55]. A set of 108 sequences was retrieved and immediately reduced by removing duplicate sequences, false positives (*i.e*. non-zinc containing proteins), and NMR structures having minimal restraints and/or domain sizes. The remaining 46 RING finger-like sequences were aligned by multiple sequence alignment; the sequences are listed in Additional file 2. The PROMALS and PROMALS3 D programs were used for all sequence alignments [56,57]. The four most closely related sequences, by sequence similarity and metal binding residue disposition, were used for homology modeling using MODELLER software [58]. PDB structures [1FBV, 3HCT, 1UR6, and 1WEO] were used to restrain the SPE42 model based on a PROMALS3 D seed alignment following N and C-terminal truncation to match sequence lengths. Functionally important residues were identified by structural alignment of the SPE-42 model with 3HCT (TRAF6 RING finger domain and its Ubc13 E2 domain partner) using Chimera [59]. Following alignment, all residues within 5Å of the E2 ubiquitin-conjugating enzyme were determined (Figure 2A and 2B).

## Authors' contributions

LDW and TLK made molecular constructs and performed worm microinjection. LDW, JMS and TLK derived transgenic worm lines. LDW, BDM, ALR, KT and TLK performed worm crosses and progeny counts. JNR performed *in silico *modelling and analysis of the RING domain and wrote the corresponding sections of the manuscript. TLK designed and supervised the experiments and wrote the remainder of the manuscript. All authors read and approved the final manuscript.

## Supplementary Material

Additional file 1 **RING finger overlay and homology model of SPE-42 RING domain**. PNG image file showing (A) Structural alignment of known RING finger proteins 1FBV (blue), 3HCT (white), 1UR6 (red) and 1WEO (orange) with putative SPE-42 RING finger (green). (B) SPE-42 model resulting from the simultaneous comparison to 1FBV, 3HCT, 1UR6, and 1WEO using MODELLER. Overall backbone structure, location of the 2 Zn^++ ^ions, and position of critical cysteines are shown. Click here for file

Additional file 2 **RING finger-like sequences used for alignment with SPE-42**. Excel spreadsheet file containing accession numbers and descriptions for the 46 RING finger proteins initially considered for modeling the SPE-42 RING domain. The top 4 proteins in the list were used to build the SPE-42 structural model. Click here for file
