# Supplementary figures and images for: Fertilization in C. elegans requires an intact C-terminal RING finger in sperm protein SPE-42
Source: BMC Dev Biol. 2011 Feb 23;11:10. doi: 10.1186/1471-213X-11-10 (PMC3053230; doi:10.1186/1471-213X-11-10)

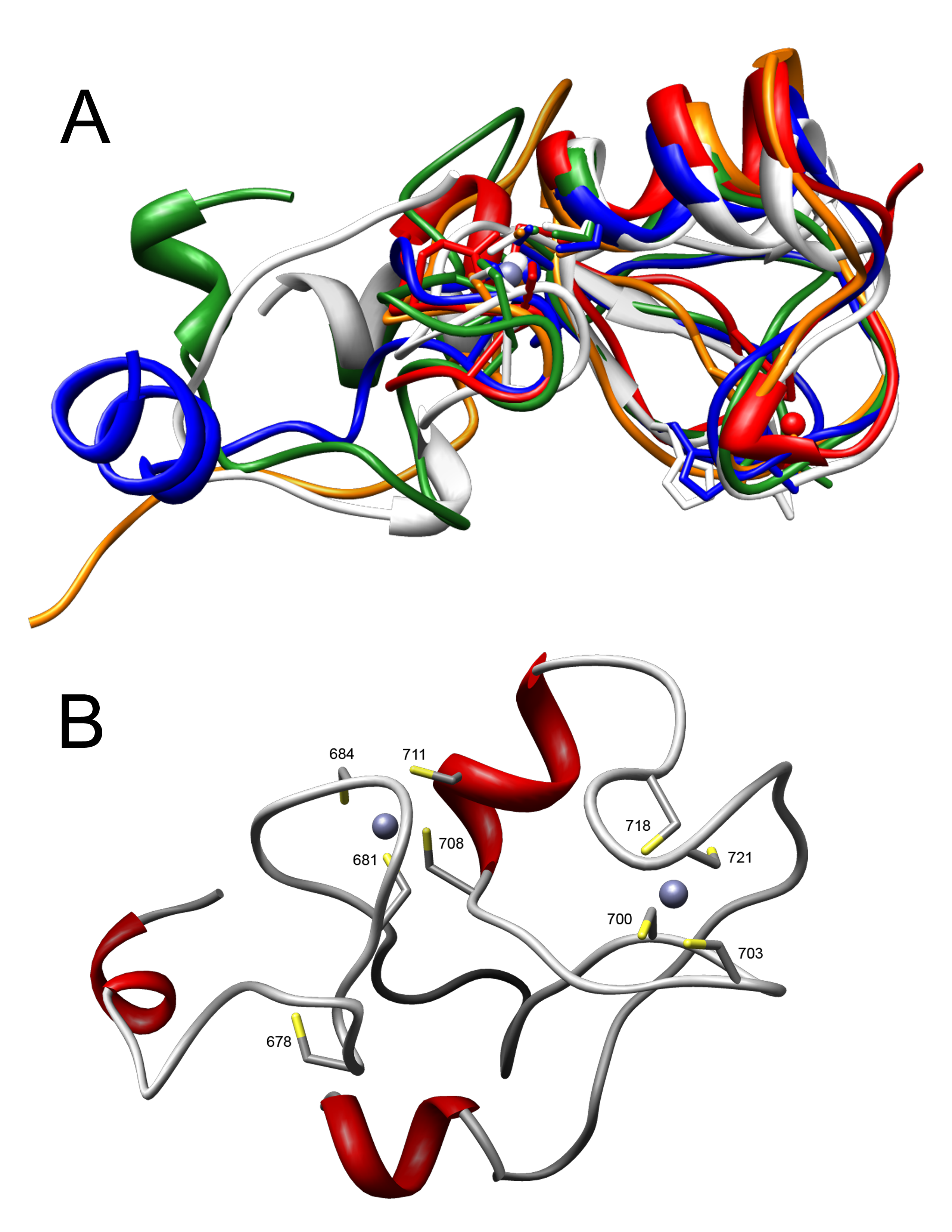

Supplement: Additional file 1 — RING finger overlay and homology model of SPE-42 RING domain. PNG image file showing (A) Structural alignment of known RING finger proteins 1FBV (blue), 3HCT (white), 1UR6 (red) and 1WEO (orange) with putative SPE-42 RING finger (green). (B) SPE-42 model resulting from the simultaneous comparison to 1FBV, 3HCT, 1UR6, and 1WEO using MODELLER. Overall backbone structure, location of the 2 Zn++ ions, and position of critical cysteines are shown. [file 1471-213X-11-10-S1.PNG]
